# Treatment of Acute Rheumatism

**Published:** 1883-03-20

**Authors:** 


### THE TREATMENT OF ACUTE RHEUMATISM.

There are few, if any, subjects more trite than this, and the saying that the disease which has the greatest number of "sure cures" is the least amenable to treatment, finds in rheumatism its aptest illustration.

Rheumatism has for ages been the battle ground of pathologists and therapeutics. An article on the subject, by Dr. Roberts Bartholow, in the Medical Record, while it contains no reference to any new drug or new method of administering any drug, nevertheless makes a very helpful division of the classes of patients who are the subjects of rheumatism. This division, if it shall prove correct, will aid very materially in the intelligent selection of drugs in the treatment of this affection. It recognizes three groups of persons who are subject to rheumatic arthritis:

1st. Spare persons of considerable bodily vigor, good muscular development, and having a distinct family history of neurotic or rheumatismal disorders.

2d. Obese subjects, addicted to malt liquors and good living, sometimes with—more often without—an inherited predisposition to rheumatic diseases; the gelatinous descendants of albuminous parents, as they have been entitled.

3d. The feeble, pale, anæmic subject, depressed by poor diet, and even hygienic surroundings, including dampness and bad air.

"No one," says Dr. Bartholow, "can treat cases of rheumatism successfully unless he recognizes the type before him, and adopts his remedies accordingly."

In the first class of cases he prescribes salicylic acid or the salicylate of soda. He has no theory to account for the action of this drug or for its superiority to others in this class of cases. In view of the special liability to relapses in this class of cases the remedy should be continued for several days after the acute symptoms have subsided.

In the second class of cases there is a tendency to a form of acid indigestion, and they are those in which the alkaline treatment has been found most valuable. In view of the diverse conceptions which obtain in regard to what constitutes the alkaline treatment, Dr. Bartholow quotes with his approval the plan as understood by Dr. Fuller, the author of an excellent work on rheumatism :

"By the 'alkaline treatment,'" says Dr. Fuller, "I mean a plan of treatment in which alkalies play an important part, but which consists not only in the administration of alkalies, but in the careful regulation of the secretions, the strictest attention to diet, and the administration of tonics, such as quinine and bark, as soon as the patient can bear them. * * * * My practice is to give not less than an ounce and a half of the alkaline carbonates, either alone or in combination with a vegetable acid, during the first twenty-four hours of treatment. * * * More commonly two drachms are ordered to be taken in effervescence every three or four hours, in combination with an ounce of lemon-juice, or with a drachm of citric acid dissolved in four ounces of water. At the same time, if the bowels are torpid, ten grains of colocynth and calomel pill (British Pharm.) are prescribed at bed-time. As soon as the urine, when freshly voided, ceases to show an acid reaction—which is usually the case after twenty-four hours—the quantity of the alkali is diminished by one-half, six drachms only being administered during the succeeding twenty-four hours. At the expiration of that time, if the urine remains alkaline, three drachms only are given in the next twenty-four hours ; and on the fourth day, if the urine still shows an alkaline reaction, the form of the medicine is altogether changed. The treatment ceases to be essentially alkaline; either a cinchona draught is ordered to be taken three times a day, containing a scruple or a half drachm of bicarbonate of potash—a little more or a little less, according to the condition of the urine, which should be kept nearly neutral—or three grains of quinine dissolved in lemon-juice is given three times a day in effervescence, with half a drachm of bicarbonate of potash or soda. * * * * * The diet is restricted to beef tea or broth, with soda-water and milk, and barley-water as a drink, as the smallest quantity of solid food, given a day before the tongue has thoroughly cleaned, is apt to induce a recurrence of the disease. Wine and spirits are strictly forbidden, though experience has convinced me that wine and spirits prove less hurtful than the smallest quantity of solid food."

The third class of cases is numerically the most important, besides being also those in which vicious results are most liable to develop, cardiac complications being relatively frequent. The depressing effects of salicylic acid and alkalines are to be avoided in these.

To Dr. Russell Reynolds, of London, is due the introduction of the most successful drug in this class of cases: Tincture of the chloride of iron. It must be given in full doses—ʒ ss to ℥ j—properly diluted, every four to eight hours. It lessens the swelling and pain in the joints, lowers the fever, diminishes the tendency to heart complications; and, above all, sustains the vital powers in their struggle against the encroachment of the rheumatic disease.

The treatment as above outlined does not prevent a resort to blisters, and indeed the author speaks of them as deserving careful consideration. It is a remarkable fact, he says, that blistering brings about a neutral or alkaline condition of the urine. The usual method of blistering is by means of cantharides, and the plans of their application have been various, ranging from a small blister over and in the neighborhood of the joint, to a complete zone encircling it. We have found in our experience that the application of a tartar emetic ointment ( ʒ j to ℥ j ) is much to be preferred to cantharides. It is more cleanly, less disagreeable to the patient, and more speedy in the relief it brings. The appearance of the characteristic pustules is usually attended by immediate amelioration of pain.—*Medical Age.*

